# Mapping the Intellectual Structure of Intercultural Intelligence in Digitally Mediated Contexts: A Bibliometric and Thematic Analysis (2006–April 2026)

**DOI:** 10.3390/jintelligence14070125

**Published:** 2026-07-01

**Authors:** Aylin Akinlar

**Affiliations:** Faculty of Communication, Bandirma Onyedi Eylul University, Bandirma 10200, Türkiye; draylinakinlar@gmail.com

**Keywords:** intercultural intelligence, cultural intelligence, digitally mediated communication, global virtual teams, bibliometric analysis, science mapping, thematic evolution, collaboration networks

## Abstract

This study presents a bibliometric and thematic analysis of intercultural intelligence research in digitally mediated contexts. Using a dataset of 92 journal articles indexed in the Web of Science Core Collection between 2006 and April 2026, the study maps the intellectual structure, collaboration patterns, and thematic evolution of the field. Data were analyzed using the Bibliometrix R package (version 5.3.0) and Biblioshiny through performance analysis, science mapping, co-citation analysis, collaboration networks, and thematic mapping techniques. The findings indicate that research output has expanded considerably since the mid-2010s, reflecting the growing importance of intercultural competencies in digitally connected environments. The intellectual structure of the field remains strongly anchored in cultural intelligence theory while increasingly incorporating themes related to communication, trust, global virtual teams, leadership, and digitally mediated collaboration. Thematic evolution analyses reveal a gradual shift from traditional cultural intelligence constructs toward broader concerns associated with virtual interaction, digitally mediated collaboration, and emerging AI-supported communication environments. The results also demonstrate a highly collaborative and internationally connected research landscape, although notable geographical imbalances remain. By providing a systematic overview of the field, this study contributes to a deeper understanding of how intercultural intelligence scholarship is evolving in response to digital transformation and offers directions for future interdisciplinary research.

## 1. Introduction

In an increasingly interconnected and digitalized world, the ability to function effectively across cultural boundaries has become a critical competence for individuals and organizations. This capability, commonly conceptualized as cultural intelligence (CQ), refers to an individual’s capacity to adapt successfully to culturally diverse environments (Earley & Ang, 2003). Building on traditional intelligence theory, cultural intelligence has been widely recognized as a multidimensional construct encompassing metacognitive, cognitive, motivational, and behavioral dimensions (Ang et al., 2007). In this study, the term intercultural intelligence is used as an umbrella concept encompassing cultural intelligence and closely related intercultural competence frameworks.

Over the past two decades, globalization has significantly intensified intercultural interactions, not only through physical mobility but also through digital communication technologies. The rise of social media, online learning environments, and artificial intelligence (AI)-mediated communication has fundamentally transformed how individuals engage with cultural diversity (Deardorff, 2006; Erez et al., 2013). These digital environments facilitate intercultural contact beyond geographical constraints while simultaneously introducing new challenges related to communication, coordination, and interpretation in virtual settings.

Within this evolving landscape, cultural intelligence has become increasingly important as a key predictor of effective intercultural communication, adaptation, and performance. Empirical studies have consistently shown that individuals with higher levels of CQ are more successful in cross-cultural adjustment, global teamwork, and leadership effectiveness (Rockstuhl et al., 2011). CQ has been linked to improved performance outcomes in culturally diverse settings, including expatriate adjustment and international collaboration (Ang et al., 2007; Earley & Mosakowski, 2004).

More recently, scholars have begun to explore the role of cultural intelligence in digitally mediated contexts. Research has examined how CQ influences virtual collaboration, online communication, and interactions within culturally diverse virtual teams (Erez et al., 2013). Despite these developments, the conceptual integration of cultural intelligence and digitalization remains limited and fragmented across disciplines.

Existing literature on cultural intelligence has largely developed within the fields of management, psychology, and intercultural communication, often focusing on specific outcomes such as job performance, leadership, or adaptation. At the same time, research on digital communication and online interaction has evolved as a separate domain, emphasizing technological affordances and user behavior. Although these streams intersect in practice, there is still a lack of systematic understanding of how cultural intelligence is conceptualized and studied within digital contexts. This fragmentation results in dispersed knowledge structures, inconsistent terminology, and limited theoretical integration.

To address this limitation, bibliometric analysis provides a robust methodological approach for systematically mapping the intellectual structure and thematic evolution of a research field (Aria & Cuccurullo, 2017, 2026). Bibliometric techniques enable the identification of influential authors, key publication sources, collaboration networks, and emerging research trends by analyzing large-scale scientific datasets (Donthu et al., 2021; Ellegaard & Wallin, 2015). Furthermore, science mapping and thematic analysis allow for a deeper understanding of how concepts cluster and evolve, revealing the underlying knowledge structure of a domain (Aria & Cuccurullo, 2017, 2026; Zupic & Čater, 2015).

Although bibliometric studies have been conducted in related areas, such as intercultural communication and digital learning, a comprehensive analysis of the intersection of cultural intelligence and digitalization remains lacking. Given the rapid expansion of digital environments and the increasing relevance of intercultural competencies in these contexts, there is a clear need for a systematic and integrative examination of this emerging research field.

Responding to this gap, the present study conducts a bibliometric and thematic analysis of 92 peer-reviewed journal articles indexed in the Web of Science (WoS) Core Collection between 2006 and April 2026. Using performance analysis, science-mapping techniques, and thematic exploration procedures, the study examines the intellectual structure, collaboration patterns, conceptual organization, and thematic evolution of intercultural intelligence research in digitally mediated contexts. By identifying influential publications, leading contributors, core research themes, and emerging directions, the study aims to provide a comprehensive overview of how the field has developed and adapted to increasingly digitalized forms of intercultural interaction.

### Previous Reviews and Bibliometric Studies

Research on cultural and intercultural intelligence has expanded considerably over the last two decades, generating a substantial body of empirical work across management, organizational behavior, communication, and education. Existing studies have primarily examined the relationships between cultural intelligence and outcomes such as performance, leadership effectiveness, cross-cultural adjustment, global teamwork, and organizational success (Ang et al., 2007; Erez et al., 2013; Taras et al., 2013). More recent scholarship has increasingly explored the role of cultural intelligence in digitally mediated environments, including virtual teams, online collaboration, and technology-enabled intercultural interaction (Hu et al., 2017; Hu et al., 2020; Presbitero, 2020).

Despite the growing volume of research, the literature remains fragmented across multiple disciplinary domains and publication outlets. Previous reviews have largely focused on conceptual development, measurement issues, or specific organizational outcomes, whereas comprehensive science-mapping studies examining the intellectual structure and thematic evolution of intercultural intelligence within digitally mediated contexts remain limited. Consequently, there is still insufficient understanding of how foundational theories, influential contributors, collaboration networks, and emerging themes are interconnected across the broader knowledge structure of the field. Addressing this gap, the present study employs bibliometric and thematic analysis techniques to provide a systematic overview of the field’s intellectual architecture, thematic development, and future research directions.

Beyond providing a descriptive overview of publication trends, this study seeks to clarify how intercultural intelligence research has been intellectually organized and transformed within digitally mediated environments. The growing prevalence of global virtual teams, digitally mediated communication, online learning environments, and emerging AI-supported communication environments has created new contexts in which intercultural competencies are enacted and evaluated. Consequently, understanding the conceptual structure, dominant themes, and evolving research trajectories of this field is important not only for scholars of intercultural intelligence but also for researchers working in communication, management, education, organizational behavior, and digital transformation. By combining performance analysis with science mapping techniques, the present study moves beyond a conventional literature review and provides a systematic examination of the field’s intellectual architecture and thematic development.

By providing a comprehensive mapping of the field, this study contributes to the literature in three ways. First, it offers a systematic overview of intercultural intelligence research in digitally mediated contexts, addressing fragmentation across disciplinary boundaries. Second, it identifies dominant and emerging themes, highlighting how digital technologies are reshaping intercultural competence and collaboration. Third, it provides a foundation for future interdisciplinary research at the intersection of intercultural intelligence, communication, and digital transformation.

Specifically, this study addresses the following research questions:

RQ1: How has research on intercultural intelligence in digitally mediated contexts evolved in terms of publication trends and key contributors?

RQ2: What does the intellectual and social structure of the field reveal through science-mapping techniques?

RQ3: What are the dominant themes, and how have they evolved within intercultural intelligence research in digitally mediated contexts?

## 2. Materials and Methods

### 2.1. Data Collection and Search Strategy

This study employed a bibliometric research design to examine the intellectual structure, thematic evolution, and collaboration patterns of research on intercultural intelligence in digitally mediated contexts. Bibliometric analyses and science mapping procedures were performed using the Bibliometrix R package (version 5.3.0) and its Biblioshiny interface, which provide comprehensive tools for performance analysis, conceptual structure mapping, thematic evolution analysis, and science mapping studies (Aria & Cuccurullo, 2017, 2026; Donthu et al., 2021). The bibliographic data were retrieved from the Web of Science (WoS) Core Collection, which is widely recognized as one of the most authoritative sources for bibliometric investigations due to its rigorous indexing standards and comprehensive coverage of peer-reviewed scholarly literature (Mongeon & Paul-Hus, 2016).

To enhance the dataset’s precision, the search strategy was refined, and the corpus was reconstructed through additional screening and validation. In the initial retrieval process, the abbreviation “CQ” was included as a search term. Because this abbreviation is used across multiple disciplines unrelated to cultural intelligence, it generated several potentially irrelevant records. To improve conceptual precision and dataset validity, the abbreviation was removed and replaced with explicit conceptual descriptors.

The final search was conducted in the Web of Science Core Collection on 9 April 2026 using the following search query:

TS = ((“cultural intelligence” OR “intercultural intelligence” OR “cultural quotient” OR “intercultural competence” OR “cross-cultural competence”) AND (“digital communication” OR “social media” OR “online learning” OR “virtual team*” OR “computer-mediated communication” OR “artificial intelligence” OR “AI-mediated communication” OR “digital environment*” OR “digitalization”)).

The search was restricted to journal articles included in the Social Sciences Citation Index (SSCI) and Emerging Sources Citation Index (ESCI). Only English-language publications were included. Following the retrieval process, all records were manually screened to identify and remove studies that were not conceptually related to intercultural intelligence in digitally mediated contexts. Particular attention was given to excluding biomedical, technical, and unrelated studies retrieved through overlapping terminology.

After the screening and refinement process, the final dataset consisted of 92 journal articles published between 2006 and April 2026. These records formed the basis of all subsequent bibliometric analyses.

### 2.2. Data Analysis Tools and Procedures

The bibliometric analyses were conducted using the Bibliometrix package (version 5.3.0) and its web-based interface Biblioshiny in R (Aria & Cuccurullo, 2017, 2026). Bibliometrix is a widely used open-source framework that enables comprehensive performance analysis, science mapping, thematic exploration, and visualization of bibliographic data. Following data collection and screening, the analytical procedure was organized into three complementary stages: performance analysis, science mapping, and thematic analysis. This multi-stage approach allowed the study to examine not only the productivity and impact of the field but also its intellectual structure, collaboration patterns, conceptual organization, and thematic evolution over time.

All bibliometric analyses, science mapping procedures, and visualizations were generated using the Bibliometrix package (version 5.3.0) and its Biblioshiny interface (Aria & Cuccurullo, 2017, 2026). These tools were employed to produce performance indicators, collaboration networks, co-occurrence maps, thematic maps, thematic evolution analyses, co-citation networks, three-field plots, and other visual representations presented throughout the study.

#### Performance Analysis

Performance analysis was conducted to evaluate the productivity, visibility, and scholarly impact of research on intercultural intelligence in digitally mediated contexts. This stage focused on identifying the main contributors, publication trends, and citation patterns that characterize the development of the field. Specifically, the analysis examined annual scientific production, average citations per year, the most productive authors, the most relevant sources, leading institutional affiliations, and country-level research output.

In addition, citation-based indicators were used to identify highly influential publications and to assess the intellectual visibility of the field. Bradford’s Law was employed to identify the core journals contributing to the knowledge base of intercultural intelligence research. According to Bradford’s Law, publications within a scientific field tend to be concentrated in a relatively small set of highly productive journals, while progressively larger groups of journals contribute fewer relevant articles (Bradford, 1934; Brookes, 1969; Egghe, 1986). Applying this principle enabled the identification of the core publication sources and the assessment of the extent to which intercultural intelligence research is concentrated within a limited group of journals or dispersed across a broader range of publication outlets.

### 2.3. Thematic Analysis and Visualization

To investigate the conceptual structure of the field, keyword co-occurrence analyses were performed using both author keywords and Keywords Plus. Co-occurrence analysis enables the identification of dominant concepts and thematic relationships by examining how frequently keywords appear together within the same publications. This approach provides insights into the conceptual organization of a research domain and helps reveal major thematic clusters.

In addition, thematic mapping was conducted based on the centrality and density measures proposed by Cobo et al. (2011). Centrality reflects the degree of interaction between a theme and other themes within the research field, whereas density indicates the internal development and cohesion of a thematic cluster. Based on these two dimensions, themes were classified into four categories: motor themes (high centrality and high density), basic themes (high centrality and low density), niche themes (low centrality and high density), and emerging or declining themes (low centrality and low density).

To examine thematic change over time, thematic evolution analysis was also performed. This technique traces the development, continuity, and transformation of major research themes across different time periods, thereby providing insights into how the conceptual focus of the field has evolved.

Finally, visualization techniques, including keyword networks, thematic maps, collaboration maps, co-citation networks, three-field plots, and word clouds, were employed to facilitate the interpretation of bibliometric findings and to provide a comprehensive representation of the intellectual, social, and conceptual structure of intercultural intelligence research in digitally mediated contexts.

### 2.4. Reliability and Validity Considerations

Several measures were implemented to enhance the reliability, validity, and reproducibility of the study. First, the Web of Science Core Collection was selected as the sole data source because of its rigorous indexing standards and widespread use in bibliometric research (Mongeon & Paul-Hus, 2016). The search strategy was designed to balance comprehensiveness and conceptual relevance by combining intercultural intelligence-related concepts with digitally mediated communication and interaction terms.

Second, following the peer-review process, the dataset underwent an additional refinement procedure to address potential corpus contamination. During the initial retrieval stage, the abbreviation “CQ” generated a small number of records from unrelated domains because the abbreviation is used across multiple disciplines. To improve dataset validity, the search strategy was revised, the abbreviation was removed, and all retrieved records were manually screened. Publications that were not conceptually related to cultural or intercultural intelligence in digitally mediated contexts were excluded from the final dataset. This refinement process increased the thematic coherence and conceptual specificity of the corpus used for analysis.

Third, the study employed established bibliometric procedures and widely recognized analytical tools, including Bibliometrix and Biblioshiny (Aria & Cuccurullo, 2017, 2026). Multiple complementary techniques, including performance analysis, co-citation analysis, collaboration analysis, thematic mapping, and thematic evolution analysis, were used to enable methodological triangulation and strengthen the robustness of the findings.

However, certain limitations should be acknowledged. The analysis was limited to English-language articles indexed in the Web of Science Core Collection, which may exclude relevant studies published in other languages or indexed in other databases. In addition, bibliometric analyses rely on metadata and citation information, which may not fully capture the qualitative depth of individual studies. Although the Web of Science Core Collection provides high-quality bibliographic metadata and citation consistency, future bibliometric studies may benefit from incorporating additional databases such as Scopus and Dimensions to improve coverage and enable database triangulation.

## 3. Results

### 3.1. Publication Trends

**Figure 1:**
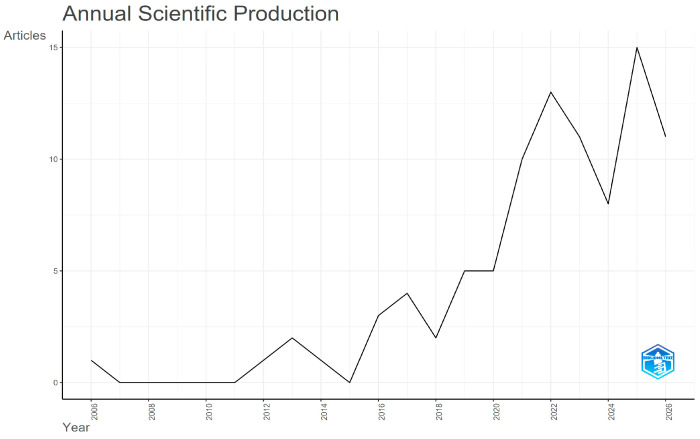
Annual scientific production.

As illustrated in Figure 1, research on intercultural intelligence in digitally mediated contexts remained highly limited during the initial phase of the field. Between 2006 and 2015, annual scientific production was sporadic, with most years recording either no publications or only one to two articles. This period reflects the early emergence of research connecting cultural intelligence with digital and technology-mediated environments.

A gradual increase became visible after 2016. Annual output rose from three publications in 2016 to five publications in both 2019 and 2020, indicating growing scholarly interest in the intersection of intercultural competencies and digitally enabled interaction. The field entered a more pronounced expansion phase after 2020. Annual production increased to 10 publications in 2021 and reached 13 publications in 2022, reflecting the growing relevance of virtual collaboration, digital communication, and technology-supported intercultural engagement.

Although publication output fluctuated slightly in subsequent years, research activity remained consistently higher than in the earlier stages of development. The highest annual production was recorded in 2025 with 15 publications. The slight decline observed in 2026 should be interpreted cautiously because indexing for the current publication year may still be incomplete. Overall, the results indicate that intercultural intelligence research in digitally mediated contexts has evolved from a niche area of inquiry into an increasingly visible and expanding research domain.

**Figure 2:**
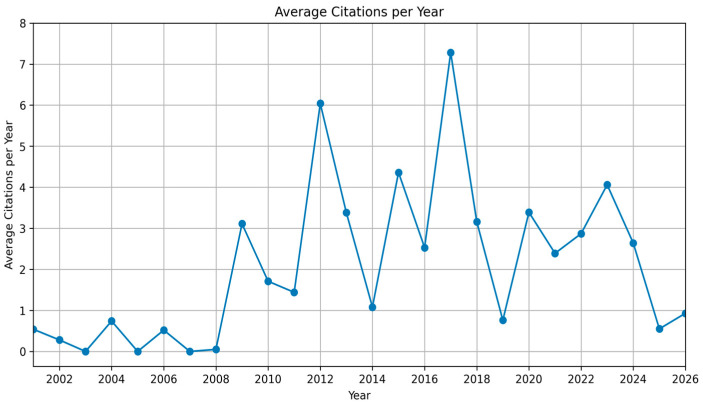
Average citations per year.

Figure 2 presents the evolution of average citations per year, providing insight into the scholarly influence of publications within the field. Citation impact remained limited during the earliest years of the dataset, reflecting the small number of publications and the emerging nature of the research area. A substantial increase is observed around 2012–2013, with average citations reaching their highest levels during this period. This pattern suggests that several influential studies published during the formative years of the field became important reference points for subsequent research.

Following this peak, citation averages fluctuated considerably across years. Moderate citation peaks are visible between 2017 and 2021, indicating continued intellectual influence while the field expanded into new thematic areas. The coexistence of publication growth and fluctuating citation patterns suggests that intercultural intelligence research has diversified conceptually rather than consolidating around a single dominant research stream.

The declining citation averages observed after 2022 should be interpreted with caution. More recent publications have had less time to accumulate citations, creating a citation-lag effect that commonly affects bibliometric analyses. Therefore, lower citation values in the most recent years do not necessarily indicate reduced scholarly impact but rather reflect the recency of the publications. Taken together, the findings suggest that the field has developed from a small and emerging research area into a growing and increasingly recognized domain characterized by both expanding productivity and sustained intellectual influence.

In addition, several recent publications associated with emerging themes such as artificial intelligence, global virtual teams, and digitally mediated communication have already begun to attract scholarly attention. Although citation accumulation remains limited due to publication recency, these themes appear to represent some of the most dynamic areas of contemporary intercultural intelligence research.

### 3.2. Most Productive Authors, Affiliations, and Influential Documents

The analysis of the most productive authors, institutional affiliations, and globally cited documents provides important insights into the intellectual leadership, institutional concentration, and knowledge diffusion patterns within the field.

#### 3.2.1. Most Productive Authors

**Figure 3:**
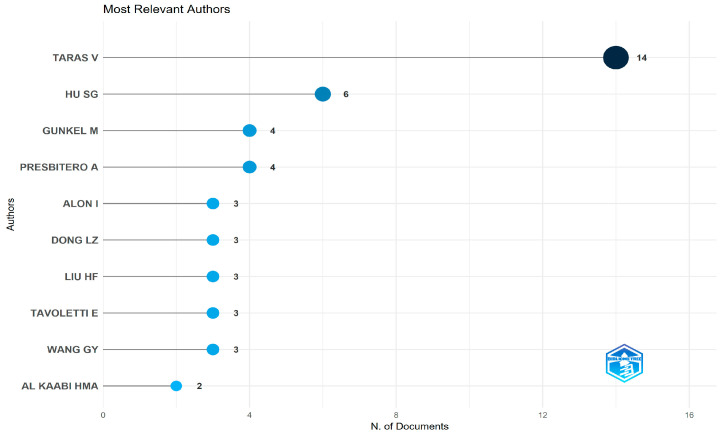
Most relevant authors.

Figure 3 presents the most productive authors in intercultural intelligence research within digitally mediated contexts. The results indicate a relatively concentrated authorship structure, with a limited number of scholars accounting for a substantial proportion of the published literature.

Among all contributors, Taras V. emerges as the most productive author with 14 publications, substantially exceeding all other researchers in the field within the retrieved dataset. This finding highlights Taras’s central role in shaping the contemporary literature on cultural intelligence, particularly through studies addressing cross-cultural interaction, global teamwork, and organizational performance.

The second most productive contributor is Hu S.G., with six publications, followed by Gunkel M. and Presbitero A., each contributing four publications. Several other scholars, including Alon I., Dong L.Z., Liu H.F., Tavoletti E., and Wang G.Y., produced three publications each, while Al Kaabi H.M.A. contributed two publications.

The distribution of authorship suggests that the field is characterized by a relatively small core of highly active researchers surrounded by a broader group of occasional contributors. Such a pattern is common in developing interdisciplinary research domains, where foundational scholars play a prominent role in establishing theoretical frameworks and research agendas. The dominance of a limited number of authors also indicates the continuing influence of cultural intelligence scholarship originally developed within management and organizational studies, while newer contributors increasingly extend these perspectives into digitally mediated environments.

Overall, the findings reveal both intellectual continuity and gradual diversification, as established scholars continue to shape the field while new researchers contribute to emerging topics related to virtual collaboration, digital communication, artificial intelligence, and intercultural interaction.

#### 3.2.2. Most Relevant Affiliations

**Figure 4:**
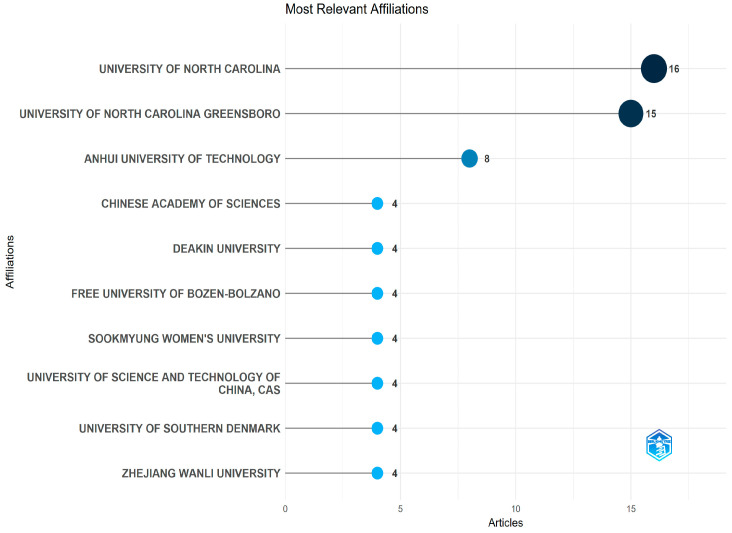
Most relevant affiliations.

Figure 4 presents the institutional affiliations that contributed most frequently to research on intercultural intelligence in digitally mediated contexts. The findings indicate a relatively concentrated institutional structure, with a small number of universities accounting for a substantial share of the scholarly output.

Among all institutions, the University of North Carolina emerged as the most productive affiliation, with 16 publications, closely followed by the University of North Carolina Greensboro, with 15 publications. This concentration suggests a concentration of research activity, reflecting their sustained engagement with cultural intelligence, cross-cultural management, and global collaboration research.

The Anhui University of Technology ranked third with eight publications, while a second group of institutions—including the Chinese Academy of Sciences, Deakin University, Free University of Bozen-Bolzano, Sookmyung Women’s University, University of Science and Technology of China, University of Southern Denmark, and Zhejiang Wanli University—each contributed four publications.

The institutional distribution reveals two noteworthy patterns. First, research activity is geographically dispersed across North America, Europe, Asia, and Australia, indicating the international character of the field. Second, the prominence of institutions with strong traditions in management, organizational behavior, international business, and intercultural studies suggests that intercultural intelligence research continues to be anchored primarily within these disciplinary domains, even as it increasingly engages with digital communication, virtual collaboration, and technology-mediated interaction.

Overall, the findings point to a field characterized by moderate institutional concentration combined with broad international participation. Such a structure reflects both the influence of established research centers and the growing diversification of scholarly contributions across different geographical and disciplinary contexts.

#### 3.2.3. Most Globally Cited Documents

**Figure 5:**
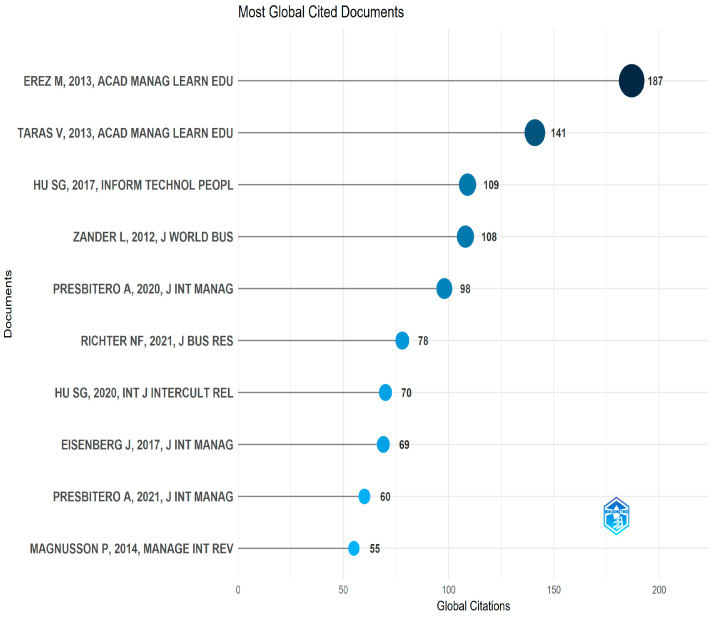
Most globally cited documents identified in the dataset: by Erez et al. (2013), Taras et al. (2013), Zander et al. (2012), Hu et al. (2017, 2020), Presbitero (2020, 2021), Eisenberg and Mattarelli (2017), Richter et al. (2021), and Magnusson et al. (2014).

Figure 5 presents the most globally cited documents within the dataset, highlighting the studies that have exerted the greatest scholarly influence on the development of intercultural intelligence research in digitally mediated contexts.

The most highly cited publication is Erez et al. (2013) with 187 global citations, followed by Taras et al. (2013) with 141 citations. Both studies represent foundational contributions to cultural intelligence research and have played a central role in shaping subsequent theoretical and empirical developments. Their prominence suggests that contemporary research continues to build upon established frameworks linking cultural intelligence to cross-cultural effectiveness, adaptation, and organizational outcomes.

Several other highly cited studies further illustrate the intellectual foundations of the field. Hu et al. (2017) received 109 citations, while Zander et al. (2012) accumulated 108 citations. These works contributed to extending cultural intelligence research toward international collaboration, global teamwork, and technology-mediated interaction. More recent influential contributions include Presbitero (2020) with 98 citations, Richter et al. (2021) with 78 citations, and Hu et al. (2020) with 70 citations, reflecting growing scholarly interest in virtual collaboration, digital communication environments, and globally distributed work settings.

The citation structure also reveals the continuing importance of research published in leading journals within management, international business, organizational behavior, and intercultural studies. Notably, several of the most influential publications appeared in journals such as *Academy of Management Learning & Education*, *Journal of International Management*, *Information Technology & People*, and *Journal of World Business*. This pattern suggests that the field remains strongly rooted in organizational and management-oriented perspectives while increasingly incorporating themes related to digital transformation and virtual interaction.

Taken together, the most highly cited documents indicate that the intellectual core of the field is anchored in cultural intelligence theory, cross-cultural adaptation, global teamwork, and organizational performance. At the same time, the presence of more recent highly cited studies focusing on virtual teams, digital communication, and technology-mediated collaboration reflects an ongoing shift toward understanding intercultural intelligence within increasingly digitalized environments.

### 3.3. Conceptual Structure of the Field

#### 3.3.1. Most Frequent Keywords and Thematic Focus

**Figure 6:**
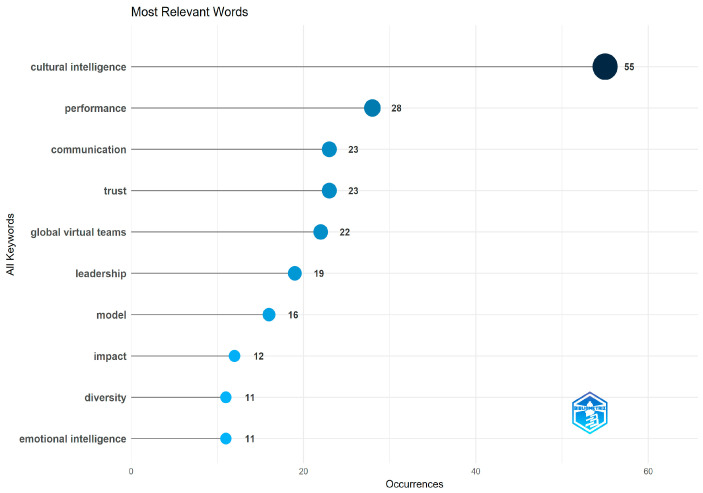
Most relevant words.

**Figure 7:**
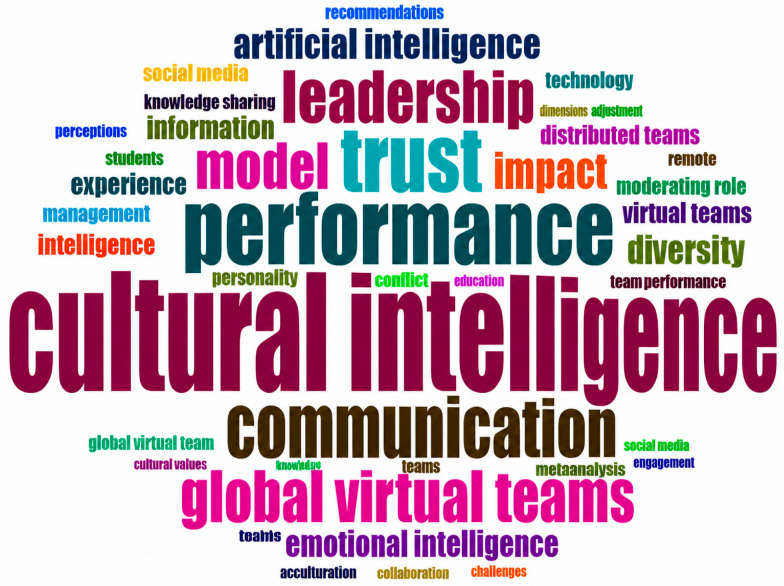
Word cloud.

Figure 6 and Figure 7 present the most frequent keywords and the word cloud generated from the refined dataset. The keyword structure indicates that the field remains strongly centered on cultural intelligence while also incorporating themes related to performance, communication, trust, leadership, diversity, emotional intelligence, and global virtual teamwork.

As shown in Figure 6, “cultural intelligence” is the most frequent keyword, appearing 55 times. This confirms its role as the central organizing concept of the field. The second most frequent term is “performance” with 28 occurrences, followed by “communication” and “trust,” each with 23 occurrences. “Global virtual teams” also appears prominently with 22 occurrences, indicating that culturally diverse and digitally mediated teamwork has become an important research context.

The prominence of “leadership” (19 occurrences), “model” (16 occurrences), “impact” (12 occurrences), “diversity” (11 occurrences), and “emotional intelligence” (11 occurrences) further demonstrates the interdisciplinary nature of the field. These terms connect intercultural intelligence research with organizational behavior, international management, communication studies, and psychology.

The word cloud in Figure 7 visually reinforces these patterns. “Cultural intelligence” occupies the largest position, while “performance,” “communication,” “global virtual teams,” “trust,” and “leadership” also appear as major thematic components. Smaller terms such as “artificial intelligence,” “distributed teams,” “virtual teams,” and “social media” suggest emerging but less dominant lines of inquiry related to digitally mediated interaction.

Overall, the keyword structure shows a conceptually coherent field. The revised dataset no longer produces unrelated biomedical or technical keywords; instead, the most frequent terms are directly aligned with intercultural intelligence, digital collaboration, communication, and organizational performance. This confirms the thematic specificity of the refined corpus and strengthens the validity of the subsequent conceptual and thematic analyses.

#### 3.3.2. Keyword Co-Occurrence and Conceptual Clusters

**Figure 8:**
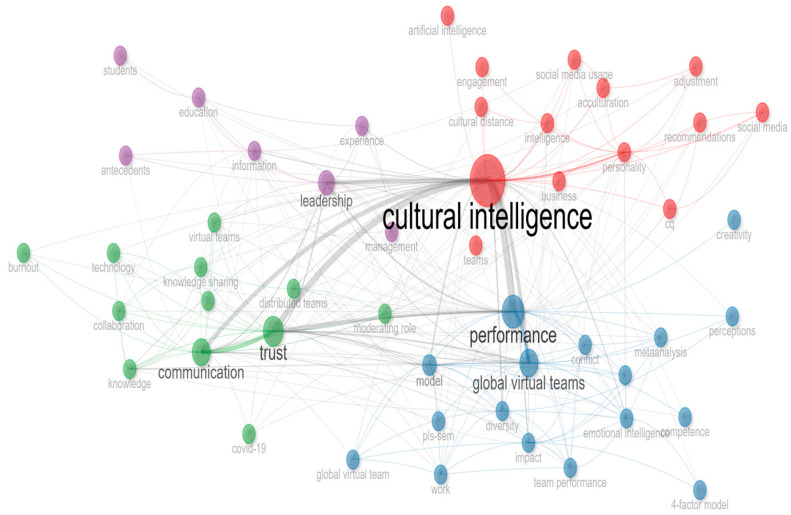
Keyword co-occurrence network. Different colors represent distinct keyword clusters identified through the network analysis, while node size reflects keyword frequency and link thickness indicates the strength of co-occurrence relationships. Visualization generated using Biblioshiny (Bibliometrix R package, version 5.3.0).

Figure 8 presents the keyword co-occurrence network generated from the author keywords and Keywords Plus included in the dataset. The network reveals the conceptual organization of intercultural intelligence research in digitally mediated contexts and highlights the relationships among the most frequently co-occurring themes.

The analysis identifies cultural intelligence as the dominant and central concept within the network, confirming its role as the field’s primary theoretical foundation. The large size and high connectivity of this node indicate that cultural intelligence functions as the intellectual hub linking multiple research streams. Closely connected keywords include performance, communication, trust, global virtual teams, and leadership, suggesting that much of the literature focuses on the practical implications of intercultural competencies in digitally mediated organizational environments.

Several thematic clusters can be observed within the network. One cluster centers on communication-related concepts, including communication, trust, and leadership, reflecting research examining how intercultural intelligence facilitates interaction and relationship-building in virtual settings. A second cluster emphasizes performance-related outcomes and global virtual teamwork, highlighting the growing interest in collaboration effectiveness within geographically dispersed teams. Additional clusters incorporate concepts associated with diversity, emotional intelligence, personality, and individual differences, indicating the interdisciplinary nature of the field and its connections with organizational psychology and intercultural communication research.

Overall, the co-occurrence structure demonstrates that intercultural intelligence research has evolved beyond the study of individual competencies alone and increasingly addresses broader organizational, technological, and communication-related challenges emerging in digitally connected environments.

#### 3.3.3. Thematic Evolution

**Figure 9:**
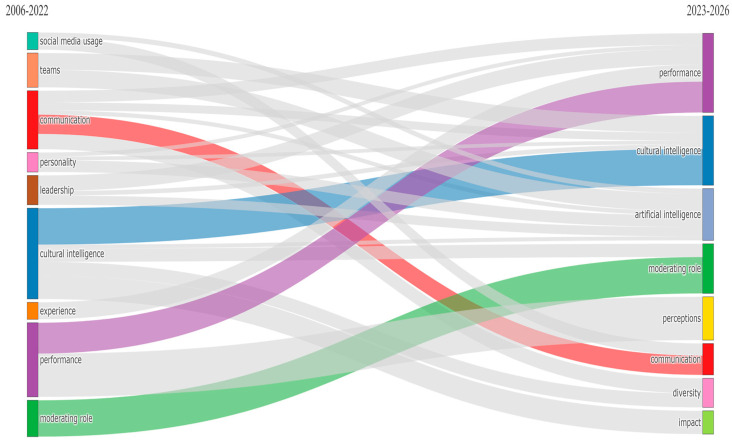
Thematic evolution of research themes, 2006–2026.

Figure 9 illustrates the thematic evolution of intercultural intelligence research across two periods: 2006–2022 and 2023–2026. The Sankey diagram reveals both continuity and transformation in the conceptual structure of the field.

During the earlier period (2006–2022), the literature was primarily organized around several foundational themes, including *cultural intelligence*, *performance*, *communication*, *leadership*, *personality*, *teams*, and *social media usage*. Among these themes, cultural intelligence and performance occupied particularly central positions, reflecting the dominant emphasis on individual capabilities and organizational outcomes.

The thematic structure observed in the later period (2023–2026) demonstrates both persistence and diversification. Cultural intelligence remains a central theme and continues to serve as the primary conceptual anchor of the field. However, new thematic connections emerge around *artificial intelligence*, indicating an increasing scholarly interest in digitally mediated intercultural interaction. Similarly, performance remains highly prominent, while communication continues to evolve as an important area of inquiry.

The emergence of themes such as *artificial intelligence*, *perceptions*, and *moderating role* suggests that recent research increasingly examines how technological environments influence intercultural competencies, communication processes, and organizational effectiveness. At the same time, the continued presence of diversity, impact, and communication-related themes demonstrates the enduring relevance of traditional intercultural research concerns.

Overall, the thematic evolution analysis indicates that the field has progressed from a relatively concentrated focus on cultural intelligence and organizational performance toward a more diversified research agenda that incorporates artificial intelligence, digital communication environments, and technology-enabled intercultural interaction.

#### 3.3.4. Thematic Map Analysis

**Figure 10:**
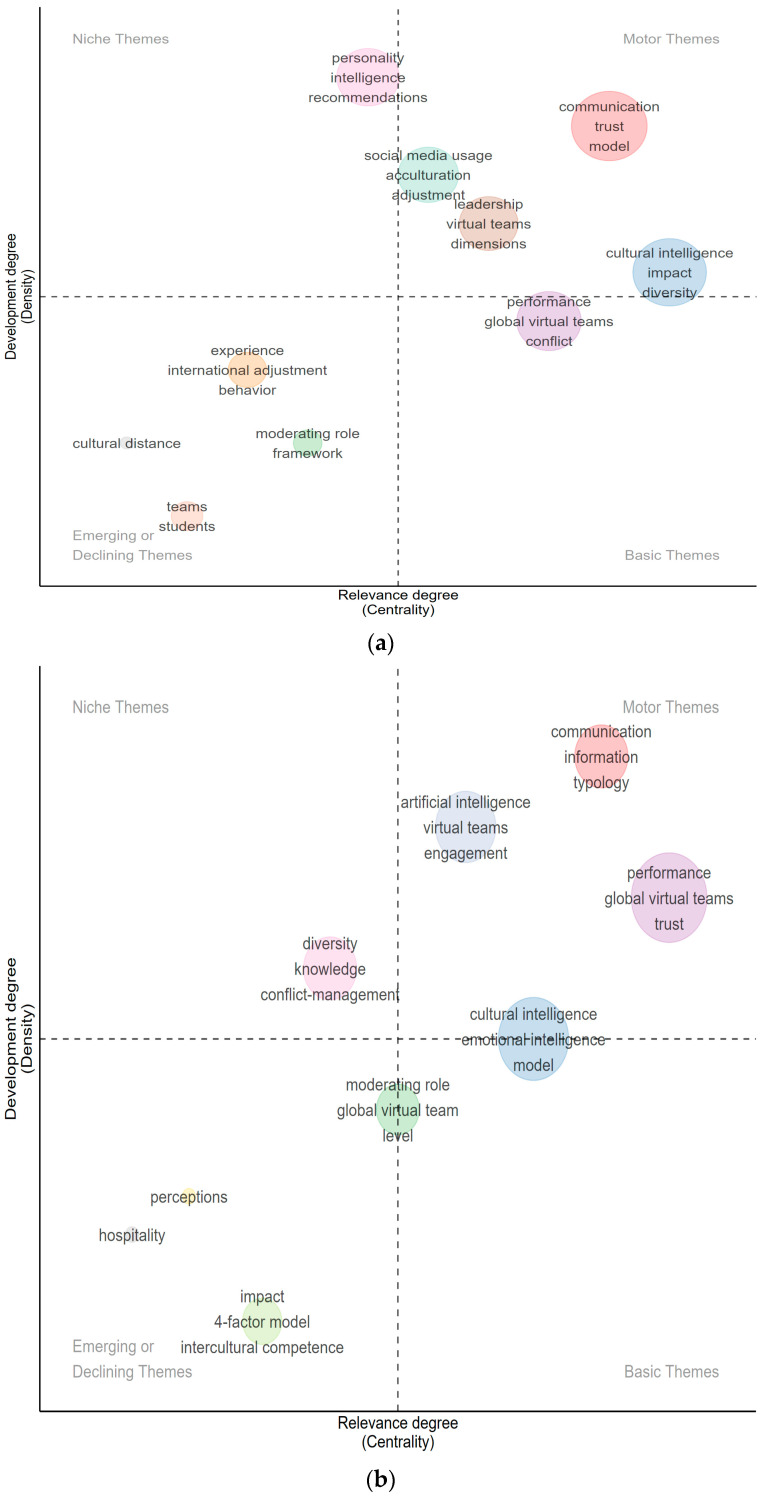
(**a**) Thematic map of intercultural intelligence research in digitally mediated contexts (2006–2022). (**b**) Thematic map of intercultural intelligence research in digitally mediated contexts (2023–2026). Bubble size represents the relative prominence of each theme, while different colors distinguish thematic clusters identified by the Bibliometrix/Biblioshiny analysis. The position of each theme is determined by centrality (x-axis) and density (y-axis). Visualization generated using Biblioshiny (Bibliometrix R package, version 5.3.0).

Figure 10a,b present the thematic structure of intercultural intelligence research in digitally mediated contexts across two consecutive periods (2006–2022 and 2023–2026). The thematic maps classify research themes according to their centrality and density, thereby revealing the degree to which particular topics are developed internally and connected to the broader knowledge structure of the field.

In the first period (2006–2022), the motor-theme quadrant was dominated by the cluster composed of *communication*, *trust*, and *model*. The location of these themes indicates that communication-related processes represented a highly developed and influential area of research. During the same period, *cultural intelligence*, together with *impact* and *diversity*, appeared as a basic theme characterized by high centrality but comparatively lower density. This positioning suggests that cultural intelligence functioned as a foundational concept linking multiple research streams while continuing to serve as the intellectual core of the field.

Several themes occupied intermediate positions between the motor and basic quadrants. These included *leadership*, *virtual teams*, and *dimensions*, reflecting increasing scholarly interest in intercultural competencies within organizational and digitally mediated work environments. In contrast, themes such as *cultural distance*, *international adjustment*, *behavior*, *teams*, and *students* appeared in the emerging or declining quadrant, indicating relatively limited integration into the broader conceptual structure of the field during this period.

The thematic structure changed noticeably in the second period (2023–2026). While *communication* remained a motor theme, it became increasingly associated with *information* and *typology*, suggesting a growing emphasis on communication processes in technologically mediated environments. A second motor-theme cluster emerged around *performance*, *global virtual teams*, and *trust*, indicating that research attention has shifted toward understanding how intercultural competencies contribute to effectiveness, collaboration, and relationship-building in virtual settings.

A particularly important development is that artificial intelligence appears in a position characterized by relatively high centrality and increasing thematic development. Although these themes are still developing, their location suggests that technological and AI-related topics are becoming increasingly integrated into intercultural intelligence research. This finding is consistent with the broader digital transformation observed across communication, management, and organizational studies.

At the same time, *cultural intelligence* continued to occupy a central position within the basic-theme quadrant and remained closely connected with *emotional intelligence* and conceptual *models*. This continuity demonstrates the enduring influence of foundational cultural intelligence theory despite the emergence of new technological themes. Meanwhile, topics such as *intercultural competence*, *4-factor model*, and *impact* appeared in the emerging or declining quadrant, suggesting either specialization into narrower research niches or reduced prominence relative to newer themes.

Taken together, the thematic maps reveal a gradual transition from traditional cultural intelligence frameworks toward a more diversified research agenda emphasizing communication, virtual collaboration, organizational performance, and artificial intelligence. The results indicate that intercultural intelligence research is increasingly adapting to the realities of digitally mediated interaction while maintaining strong connections to its original theoretical foundations.

### 3.4. Social Structure of the Field

#### 3.4.1. International Collaboration Patterns

**Figure 11:**
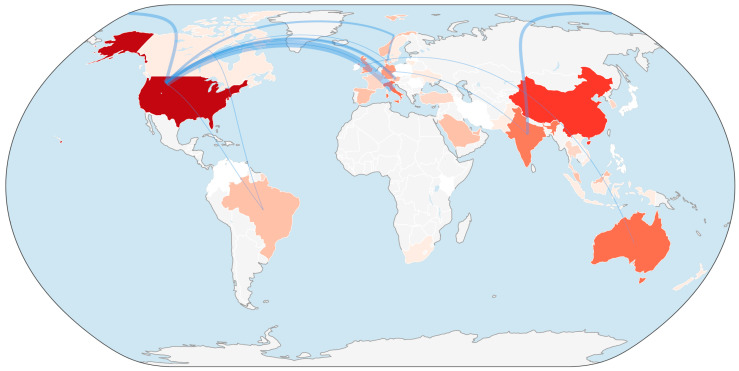
International collaboration patterns. Darker colors indicate countries with higher levels of international scientific collaboration, whereas lighter colors represent lower levels of collaboration. Visualization generated using Biblioshiny (Bibliometrix R package, version 5.3.0).

The international collaboration map (Figure 11) illustrates the global structure of research partnerships within the field of cultural intelligence and digitally mediated intercultural interaction. The results reveal a geographically diverse but uneven collaboration network. The United States occupies a central position in the global collaboration structure and demonstrates extensive research connections with scholars from Europe, Asia, and Oceania. Strong collaborative links are also visible among China, India, Australia, and several European countries, indicating the growing internationalization of research on cultural intelligence and virtual collaboration.

The distribution of collaborative ties suggests that knowledge production is concentrated in a limited number of highly connected countries that function as hubs within the global research network. At the same time, the presence of cross-continental partnerships reflects the inherently international nature of cultural intelligence research, which frequently addresses intercultural interaction, global mobility, multinational organizations, and virtual teamwork. The increasing involvement of Asian countries, particularly China and India, further indicates the expanding geographical reach of the field and the diversification of its scholarly contributors.

#### 3.4.2. Author Collaboration Network

**Figure 12:**
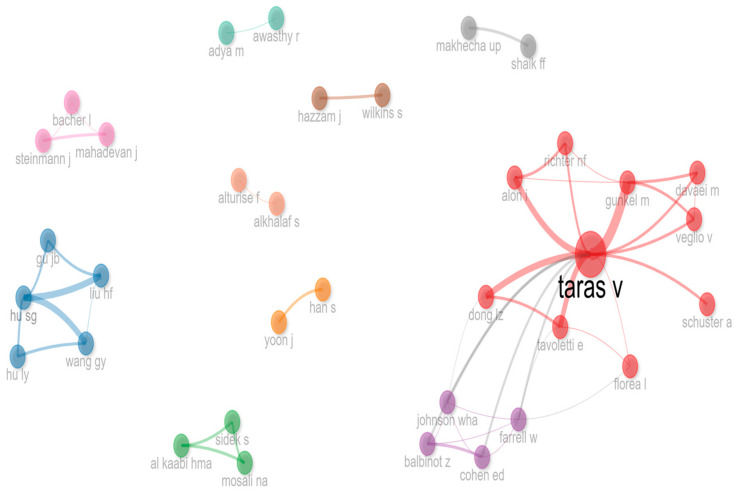
Author collaboration network. Different colors represent distinct author collaboration clusters identified through network analysis. Node size is proportional to author productivity or collaboration strength, while links indicate co-authorship relationships. Visualization generated using Biblioshiny (Bibliometrix R package, version 5.3.0).

Figure 12 presents the author’s collaboration network and highlights the social organization of scholarly production in the field. Several distinct collaboration clusters can be observed, indicating that research activity is organized around relatively stable groups of collaborating scholars rather than a single unified research community.

The largest collaboration cluster is centered on highly productive and influential authors, reflecting sustained co-authorship relationships and shared research agendas. Additional clusters represent specialized research communities focusing on specific aspects of cultural intelligence, including organizational behavior, virtual teamwork, intercultural communication, leadership, and performance. Although multiple collaboration groups are evident, the network also contains bridging connections between clusters, suggesting ongoing knowledge exchange across different thematic areas.

Overall, the collaboration structure indicates a moderately interconnected field characterized by both specialization and international scholarly cooperation. The existence of several well-defined research clusters reflects intellectual diversity while simultaneously demonstrating the cumulative development of knowledge through collaborative research practices.

### 3.5. Intellectual Structure of the Field

#### 3.5.1. Core Sources and Bradford’s Law

**Figure 13:**
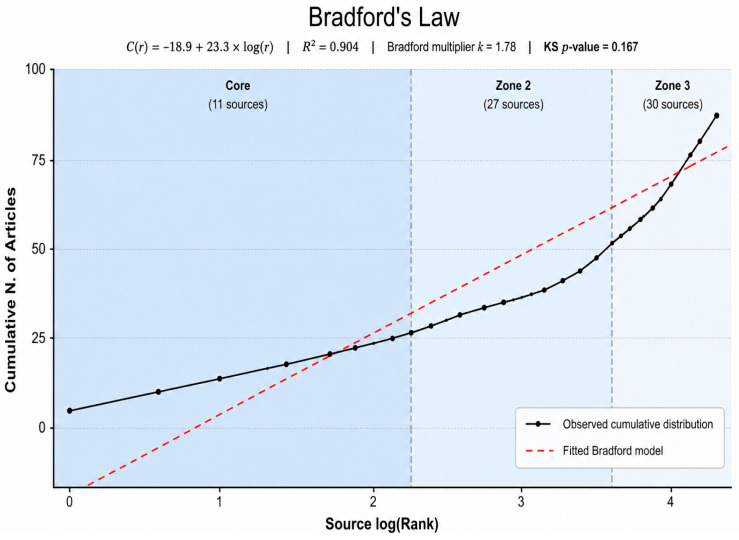
Bradford’s law distribution of sources. The solid black curve represents the observed cumulative distribution of articles, whereas the dashed red line represents the fitted Bradford model. The shaded areas indicate the Bradford zones (Core, Zone 2, and Zone 3). Visualization generated using Biblioshiny (Bibliometrix R package, version 5.3.0).

Figure 13 presents the distribution of publication sources according to Bradford’s Law and provides insight into the concentration of scholarly communication within the field. The results reveal a clear core–periphery structure. A relatively small core consisting of 11 journals accounts for a substantial proportion of the published studies, while the remaining literature is distributed across a much larger number of sources.

Table Bradford multiplier (k = 1.78) indicates a moderate level of source concentration, suggesting that knowledge production is neither excessively fragmented nor highly centralized. The second zone contains 27 sources, whereas the third zone includes 30 sources, demonstrating the progressive dispersion of publications across a wider range of journals. The fitted Bradford model exhibits a strong explanatory capacity (R^2^ = 0.904), indicating a close correspondence between the observed distribution and the theoretical expectations of Bradford’s Law.

These findings suggest that research on cultural intelligence and digital interaction is supported by a recognizable set of core journals that function as primary venues for scholarly dissemination. At the same time, the presence of numerous peripheral journals reflects the interdisciplinary nature of the field, which draws contributions from management, communication, psychology, intercultural studies, information technology, and organizational behavior.

#### 3.5.2. Co-Citation Network and Intellectual Foundations

**Figure 14:**
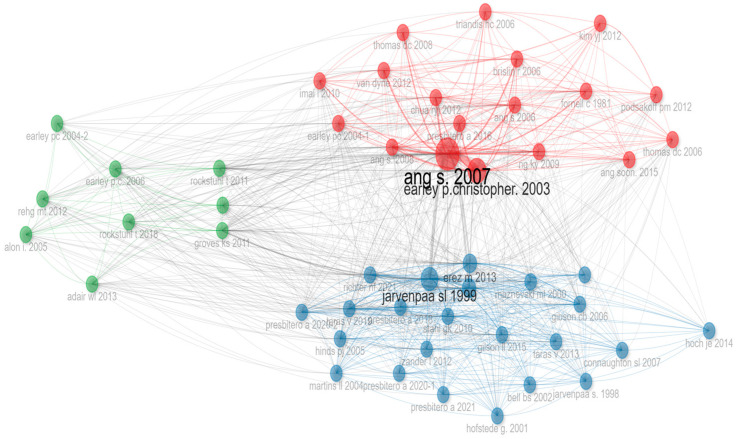
Co-citation network of references. Different colors represent distinct co-citation clusters identified through the network analysis. Node size reflects the relative citation influence of each reference, while links indicate co-citation relationships between references. Visualization generated using Biblioshiny (Bibliometrix R package, version 5.3.0).

Figure 14 illustrates the co-citation structure of the field and reveals the intellectual foundations upon which contemporary research has been constructed. The network is organized into three major clusters, indicating the existence of interconnected but distinguishable streams of scholarship.

The largest and most central cluster is dominated by foundational works associated with the development of Cultural Intelligence theory. Seminal contributions by Ang et al. (2007), Earley and Ang (2003), and related conceptual studies occupy highly central positions within the network, reflecting their role as core intellectual references across a wide range of subsequent publications. The size and density of this cluster indicate that cultural intelligence remains the primary theoretical framework guiding research in the field.

A second cluster is primarily associated with organizational and managerial applications of cultural intelligence. This stream includes studies examining leadership, organizational behavior, international management, global mobility, and workplace performance. The strong internal connectivity of this cluster suggests a relatively mature body of literature with established theoretical and empirical traditions.

The third cluster encompasses research focused on intercultural interaction, communication processes, and virtual collaboration environments. Although smaller in size, this cluster serves as an important bridge between traditional cultural intelligence research and emerging studies addressing digitally mediated communication and virtual teamwork.

Overall, the co-citation structure demonstrates a relatively cohesive intellectual foundation centered on cultural intelligence theory while simultaneously incorporating perspectives from management, communication, and organizational research. The extensive interconnections among clusters indicate substantial knowledge exchange and intellectual integration across related research domains.

#### 3.5.3. Relationships Between Influential References, Authors, and Themes

**Figure 15:**
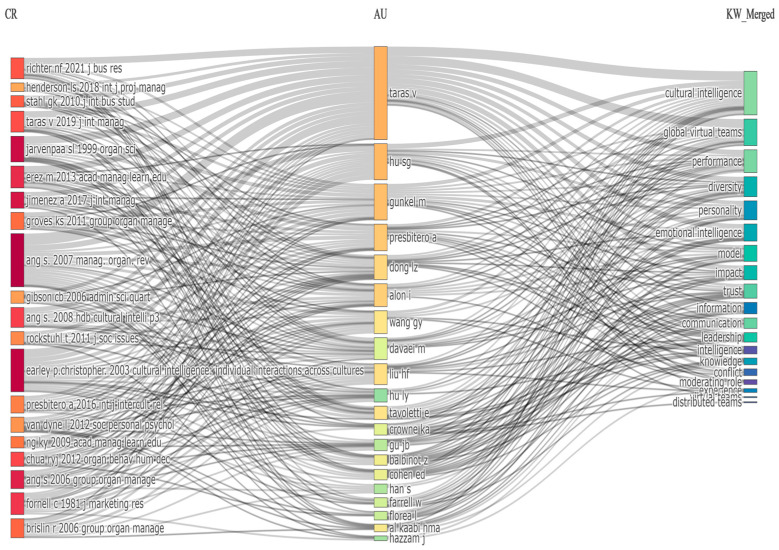
Three-field plot linking cited references (CR), authors (AU), and keywords (KW_Merged). The left column represents the most influential cited references, the middle column shows the most productive authors, and the right column presents the most frequent keywords. The connecting lines illustrate the relationships among cited references, authors, and research themes. References shown in the figure include Earley and Ang (2003), Ang et al. (2007), Jarvenpaa and Leidner (1999), Erez et al. (2013), Taras et al. (2019), Presbitero (2016), Gibson and Gibbs (2006), Rockstuhl et al. (2011), and other highly cited studies. Visualization generated using Biblioshiny (Bibliometrix R package, version 5.3.0).

The three-field plot presented in Figure 15 provides a comprehensive visualization of the relationships among influential references, productive authors, and dominant research themes. The analysis highlights the central role of several foundational publications that continue to shape contemporary scholarly discourse.

Among the cited references, seminal studies by Ang et al. (2007), Earley and Ang (2003), Erez et al. (2013), and Jarvenpaa and Leidner (1999) demonstrate strong connections with highly productive authors and frequently occurring research themes. These references form the intellectual backbone of the field and continue to influence subsequent theoretical and empirical developments.

On the author side, Taras, Hu, Gunkel, Presbitero, Dong, and Alon emerge as highly connected scholars whose work links foundational literature to contemporary research agendas. Their publications serve as important transmission mechanisms through which established theories are extended and applied to new contexts.

The keyword field reveals that cultural intelligence, global virtual teams, performance, diversity, trust, communication, emotional intelligence, and leadership constitute the principal thematic pillars of the literature. The strong linkages among references, authors, and keywords suggest a relatively coherent knowledge structure in which theoretical foundations, scholarly contributors, and research themes remain closely interconnected.

Taken together, the three-field analysis confirms that the field has developed around a stable intellectual core while simultaneously expanding toward emerging themes related to digital communication, virtual collaboration, and technology-mediated intercultural interaction.

## 4. Discussion

This study examined the intellectual, conceptual, and social structure of research on intercultural intelligence in digitally mediated contexts between 2006 and 2026. The findings reveal a field characterized by strong theoretical continuity, increasing engagement with emerging digital technologies, and expanding interest in virtual forms of intercultural interaction. At the same time, the results indicate that research activity remains concentrated around a limited number of influential authors, institutions, and countries, suggesting both intellectual consolidation and structural asymmetries in knowledge production.

### 4.1. Enduring Centrality of Cultural Intelligence Theory

One of the most consistent findings across the analyses is the continued dominance of cultural intelligence as the primary theoretical foundation of the field. Cultural intelligence emerged as the most frequently occurring keyword and occupied a central position across the co-occurrence network, thematic maps, and co-citation structure. Seminal contributions by Earley and Ang (2003), Ang et al. (2007), and subsequent theoretical developments continue to serve as the principal intellectual reference points guiding contemporary research.

The co-citation analysis demonstrates that these foundational studies remain highly interconnected with more recent publications, suggesting that the field has achieved a substantial degree of theoretical consolidation. Rather than replacing earlier frameworks, newer studies largely extend, refine, or apply existing CQ models to emerging contexts. This continuity reflects the robustness of the cultural intelligence construct and its adaptability across organizational, educational, and communication settings.

At the same time, the persistence of a relatively stable theoretical core raises questions regarding conceptual diversification. Although the field has expanded considerably, much of this growth continues to occur within the boundaries of established CQ frameworks. Consequently, future scholarship may benefit from greater engagement with alternative perspectives drawn from critical intercultural communication, digital sociology, media studies, and postcolonial approaches to intercultural interaction.

### 4.2. Digitalization and the Transformation of Intercultural Interaction

A second major finding concerns the growing integration of digitalization-related themes into intercultural intelligence research. The thematic evolution analysis demonstrates a clear transition from earlier emphases on communication, teams, leadership, and personality toward newer themes such as artificial intelligence, virtual teams, global virtual teams, performance, and digitally mediated communication.

This shift reflects broader transformations in the nature of intercultural interaction. As professional, educational, and social activities increasingly occur through digital platforms, intercultural competence is no longer confined to face-to-face encounters. Instead, individuals frequently navigate cultural differences through virtual environments, online collaboration platforms, social media networks, and AI-supported communication systems.

The thematic maps further suggest that communication, trust, and performance have emerged as highly developed motor themes. These findings indicate that contemporary research increasingly focuses on the mechanisms through which cultural intelligence contributes to effectiveness in virtual and technologically mediated environments. The prominence of global virtual teams is particularly noteworthy, as it reflects the growing relevance of remote collaboration and geographically dispersed work arrangements in the post-pandemic era.

Importantly, artificial intelligence appears as an emerging but increasingly central theme. Although AI has not yet displaced traditional cultural intelligence frameworks, its appearance within the thematic evolution structure suggests the beginning of a new research trajectory. Future studies may therefore explore how AI-mediated communication, algorithmic decision-making, and intelligent systems influence intercultural understanding, trust formation, and cross-cultural collaboration.

### 4.3. Expanding Beyond Individual-Level Perspectives

Historically, cultural intelligence research has focused predominantly on individual capabilities, including cognitive, motivational, behavioral, and metacognitive dimensions. The present findings indicate that this individual-level orientation remains influential but is increasingly complemented by broader organizational and relational concerns.

Keywords such as communication, trust, diversity, leadership, and performance occupy prominent positions within the conceptual structure. These themes suggest growing recognition that intercultural effectiveness emerges not solely from individual competencies but also from social relationships, organizational processes, and collaborative environments.

The thematic analyses further demonstrate that intercultural intelligence is increasingly examined within collective settings such as virtual teams and multinational organizations. This shift is consistent with recent scholarship emphasizing contextual and interactional dimensions of intercultural competence (Deardorff, 2006; Spitzberg & Changnon, 2009). Rather than viewing cultural intelligence exclusively as an individual attribute, contemporary research increasingly conceptualizes it as a dynamic capability operating within complex social and technological systems.

### 4.4. Internationalization and Persistent Structural Concentration

The social structure analyses reveal an increasingly international research landscape. Collaboration networks extend across multiple regions, and strong connections are visible among North America, Europe, Asia, and Oceania. Such patterns are consistent with the inherently global nature of intercultural intelligence research and reflect the transnational contexts in which cultural interaction occurs.

Nevertheless, the results also indicate a considerable degree of structural concentration. The United States and China occupy particularly prominent positions within international collaboration networks, while a relatively small group of institutions and scholars account for a substantial share of research production. The University of North Carolina and the University of North Carolina Greensboro emerge as leading institutional contributors, and authors such as Taras and Hu demonstrate particularly high levels of productivity and influence.

This concentration mirrors broader patterns observed in global scientific production and raises important questions regarding the diversity of perspectives represented within the literature. Research originating from developing regions remains comparatively limited despite the relevance of intercultural interaction in these contexts. Expanding participation from underrepresented regions may therefore enhance both the empirical richness and theoretical diversity of the field.

### 4.5. Intellectual Consolidation and Interdisciplinary Expansion

The Bradford’s Law analysis reveals a relatively concentrated publication structure characterized by a limited core of highly influential journals surrounded by a broader periphery of disciplinary outlets. This pattern suggests that the field has developed recognizable publication venues while simultaneously maintaining strong interdisciplinary connections.

The co-citation network further demonstrates the existence of a coherent intellectual foundation linking cultural intelligence, organizational behavior, intercultural communication, and international management. Rather than existing as isolated scholarly traditions, these areas appear strongly interconnected through shared references and overlapping research agendas.

The three-field analysis reinforces this interpretation by illustrating close relationships among influential references, productive authors, and dominant research themes. Cultural intelligence, performance, trust, diversity, communication, and global virtual teams function as key conceptual bridges connecting foundational scholarship with emerging research directions. As a result, the field appears both intellectually stable and sufficiently flexible to incorporate new theoretical developments arising from digital transformation.

### 4.6. Contributions of the Study

This study makes several contributions to the literature. First, it provides an updated bibliometric overview of intercultural intelligence research spanning two decades of scholarly development. Second, it identifies how digitalization, virtual collaboration, and artificial intelligence are reshaping the conceptual landscape of the field. Third, it demonstrates the coexistence of theoretical continuity and thematic innovation, revealing how established cultural intelligence frameworks continue to structure research while simultaneously accommodating emerging technological developments.

Overall, the findings suggest that intercultural intelligence research is entering a new phase of development in which digital communication technologies, virtual collaboration environments, and AI-supported interactions are becoming increasingly important. Future research should continue to investigate how these transformations influence intercultural competence, communication processes, and collaborative effectiveness across diverse cultural contexts.

## 5. Conclusions, Practical Implications, and Future Research

### 5.1. Conclusions

This study mapped the intellectual, conceptual, and social structure of research on intercultural intelligence in digitally mediated contexts between 2006 and 2026. Drawing on bibliometric performance indicators, science mapping techniques, thematic evolution analysis, and network visualization, the study provides a comprehensive overview of how the field has developed during the last two decades.

The findings indicate that intercultural intelligence research has evolved into a relatively mature and theoretically consolidated domain. Cultural intelligence remains the dominant conceptual foundation of the field, occupying a central position across keyword networks, thematic structures, and co-citation patterns. Foundational works by Earley and Ang (2003), Ang et al. (2007), and subsequent contributors continue to shape contemporary scholarship, demonstrating the enduring influence of CQ-based frameworks.

At the same time, the results reveal substantial thematic diversification. While earlier research focused primarily on communication, teams, leadership, personality, and intercultural adjustment, more recent studies increasingly address global virtual teams, trust, performance, artificial intelligence, and digitally mediated collaboration. The thematic evolution analysis suggests that the field is gradually extending beyond traditional face-to-face intercultural interaction and adapting to the realities of digital communication and virtual work environments.

The findings also demonstrate that research production remains concentrated around a limited number of influential authors, institutions, and countries. Although international collaboration networks have expanded considerably, scholarly influence continues to be concentrated within a relatively small group of academic hubs. This pattern reflects both the growing internationalization of the field and the persistence of structural asymmetries in global knowledge production.

From an educational perspective, the findings suggest that intercultural intelligence is increasingly relevant not only in organizational settings but also in digitally mediated learning environments. As universities expand internationalization efforts and online collaboration opportunities, intercultural intelligence may be viewed as a key competence that supports effective communication, collaboration, and adaptation across cultural boundaries. Consequently, future studies may investigate how intercultural intelligence can be fostered through curriculum design, intercultural learning experiences, and technology-enhanced educational practices. Overall, the study suggests that intercultural intelligence research is entering a new phase of development characterized by the coexistence of theoretical continuity and technological transformation. Rather than replacing established CQ frameworks, emerging digital themes appear to be extending and reshaping existing understandings of intercultural effectiveness in contemporary societies.

### 5.2. Practical Implications

The findings have several implications for higher education institutions, organizations, and policymakers operating in increasingly digital and culturally diverse environments.

For Higher Education

Universities and educational institutions should recognize that intercultural competence is increasingly enacted through digital environments. Educational programs may therefore benefit from integrating digital intercultural communication, virtual collaboration skills, and AI-mediated interaction into existing curricula. Beyond traditional intercultural training, students should be prepared to communicate effectively across cultures within online learning environments, international virtual exchanges, and globally connected digital platforms.

For Organizations

The growing prominence of global virtual teams, trust, communication, and performance highlights the importance of cultural intelligence in contemporary workplaces. Organizations operating across national and cultural boundaries should incorporate CQ-related competencies into leadership development, talent management, and professional training initiatives. The findings suggest that cultural intelligence remains a valuable resource for improving collaboration, trust-building, and effectiveness within remote and hybrid work environments.

For Policymakers

The increasing role of digital communication technologies in intercultural interaction has implications for policies related to education, workforce development, and international cooperation. Policymakers should support initiatives that promote digital inclusion, intercultural engagement, and equitable participation in global knowledge networks. Such efforts may contribute to strengthening social cohesion and fostering more inclusive forms of global collaboration.

### 5.3. Future Research Directions

Several directions for future research emerge from the present findings.

Digitalization and Artificial Intelligence

The thematic evolution analysis indicates that artificial intelligence is becoming increasingly visible within the field. Future studies should investigate how AI-supported communication systems, algorithmic mediation, generative AI technologies, and intelligent digital platforms influence intercultural interaction, trust formation, and collaborative performance. Greater theoretical integration between cultural intelligence and digital transformation frameworks appears particularly necessary.

Virtual Collaboration and Global Teams

The prominence of global virtual teams, communication, and performance suggests that digitally mediated collaboration will remain a major research area. Future research should examine how cultural intelligence operates within remote work environments, distributed teams, and hybrid organizational structures. Longitudinal studies may be especially valuable for understanding how intercultural competencies develop and function over time in virtual settings.

Expanding Theoretical Perspectives

Although cultural intelligence remains the dominant framework, future scholarship may benefit from greater theoretical pluralism. Perspectives derived from intercultural communication, media studies, digital sociology, critical cultural studies, and platform research could enrich existing understandings of intercultural interaction in digitally mediated contexts. Such approaches may also help address questions related to power, representation, identity, and technological mediation.

Increasing Global Diversity in Knowledge Production

The concentration of research output among a limited number of countries and institutions suggests the need for broader geographical representation. Future studies should seek to incorporate perspectives from underrepresented regions and contexts, particularly from the Global South. Expanding international collaborations and multilingual research initiatives may contribute to a more inclusive and globally representative body of knowledge.

Methodological Development

While bibliometric methods provide valuable macro-level insights into the structure and evolution of research fields, future studies may complement these approaches through qualitative, mixed-method, and experimental designs. Combining large-scale mapping techniques with context-rich empirical investigations would provide a deeper understanding of how intercultural intelligence functions in contemporary digital environments.

In conclusion, the future development of intercultural intelligence research will likely depend on its ability to integrate technological transformation with established theoretical foundations. As digital communication, virtual collaboration, and artificial intelligence continue to reshape intercultural interaction, the field has an opportunity to develop more comprehensive and context-sensitive frameworks capable of addressing the complexities of intercultural engagement in the twenty-first century.

## Data Availability

The data presented in this study are available upon request from the author.

## Abbreviations

The following abbreviations are used in this manuscript:

CQ	Cultural Intelligence
WoS	Web of Science
